# A Model of iPSC-Derived Macrophages with *TNFAIP3* Overexpression Reveals the Peculiarities of TNFAIP3 Protein Expression and Function in Human Macrophages

**DOI:** 10.3390/ijms241612868

**Published:** 2023-08-16

**Authors:** Olga Sheveleva, Elena Protasova, Tatiana Nenasheva, Nina Butorina, Victoria Melnikova, Tatiana Gerasimova, Olga Sakovnich, Alexander Kurinov, Elena Grigor’eva, Sergey Medvedev, Irina Lyadova

**Affiliations:** 1 Laboratory of Cellular and Molecular Basis of Histogenesis, Koltzov Institute of Developmental Biology of Russian Academy of Sciences, Vavilova Str., 26, 119334 Moscow, Russia; on_sheveleva@mail.ru (O.S.); elenaprotasov@gmail.com (E.P.); dreminat@mail.ru (T.N.); nnbut@mail.ru (N.B.); pochta.gerasimova@gmail.com (T.G.); olash@list.ru (O.S.); 2 Laboratory of Comparative Developmental Physiology, Koltzov Institute of Developmental Biology of Russian Academy of Sciences, Vavilova Str., 26, 119334 Moscow, Russia; v_melnikova@mail.ru; 3 Laboratory of Regeneration Problems, Koltzov Institute of Developmental Biology of Russian Academy of Sciences, Vavilova Str., 26, 119334 Moscow, Russia; alexkur@rambler.ru; 4 Laboratory of Developmental Epigenetics, Institute of Cytology and Genetics, Siberian Branch of Russian Academy of Sciences, Lavrentyev Ave., 10, 630090 Novosibirsk, Russia; evlena@bionet.nsc.ru (E.G.); medvedev@bionet.nsc.ru (S.M.)

**Keywords:** induced pluripotent stem cells, macrophages derived from induced pluripotent stem cells, A20, TNFAIP3, inflammatory response, doxycycline-inducible gene expression

## Abstract

Macrophages play a crucial role in the development and control of inflammation. Understanding the mechanisms balancing macrophage inflammatory activity is important to develop new strategies for treating inflammation-related diseases. TNF-α-induced protein 3 (TNFAIP3, A20) is a negative regulator of intracellular inflammatory cascades; its deficiency induces hyper-inflammatory reactions. Whether A20 overexpression can dampen macrophage inflammatory response remains unclear. Here, we generated human-induced pluripotent stem cells with tetracycline-inducible A20 expression and differentiated them into macrophages (A20-iMacs). A20-iMacs displayed morphology, phenotype, and phagocytic activity typical of macrophages, and they displayed upregulated A20 expression in response to doxycycline. A20 overexpression dampened the A20-iMac response to TNF-α, as shown by a decreased expression of *IL1B* and *IL6* mRNA. A dynamic analysis of A20 expression following the generation of A20-iMacs and control iMacs showed that the expression declined in iMacs and that iMacs expressed a lower molecular weight form of the A20 protein (~70 kDa) compared with less differentiated cells (~90 kDa). A low-level expression of A20 and the predominance of a low-molecular-weight A20 form were also characteristic of monocyte-derived macrophages. The study for the first time developed a model for generating macrophages with an inducible expression of a target gene and identified the peculiarities of A20 expression in macrophages that likely underlie macrophage preparedness for inflammatory reactivity. It also suggested the possibility of mitigating inflammatory macrophage responses via A20 overexpression.

## 1. Introduction

Macrophages play a crucial role in the control of host homeostasis. The function is mediated through macrophage capacity to directly eliminate pathogens, malignant and dead cells, as well as via the induction of the protective responses of other immune cells [1,2,3,4]. Different types of macrophage activities are closely related to the development of inflammatory reactions [5]. The latter, however, need to be carefully controlled in order to prevent unwanted hyperinflammatory reactions and tissue damage. It is important to understand the mechanisms that balance macrophage inflammatory activity and to learn how to target exacerbated inflammation since the latter underlies many pathologies, including chronic inflammation, autoimmune diseases, and infections [1,6,7,8].

TNF-α-induced protein 3 (TNFAIP3, also known as A20) is a cytoplasmic protein that is expressed at a low level in most cells in steady state conditions. The expression of the protein is rapidly upregulated in response to inflammatory stimuli, such as TNF-α, TLR ligands, IL-1β, or CD40L, and it serves to negatively regulate and control inflammatory responses [9,10,11,12,13]. Structurally, A20 consists of the N-terminus ovarian tumor (OUT) domain and seven zinc fingers (ZNF1-ZNF7). The OUT domain is responsible for K63 deubiquitination, ZNF4 exhibits K48 ubiquitinating activity, and ZNF7 binds linear polyubiquitin [12,13,14,15]. The ubiquitin editing activities of A20 interfere with several inflammatory signaling cascades, and they have been shown to inhibit the following: the nuclear translocation of Nuclear Factor NF-Kappa-B (NF-kB) [13,16,17], the activation of NLR Family Pyrin Domain Containing 3 (NLRP3) [17,18], the activation of interferon-regulatory factors (IRFs) induced by the retinoic acid-inducible gene I (RIG1) [19], JAK/STAT signaling [20], as well as cell necroptosis and apoptosis [21,22]. A20 was also reported to stimulate autophagy, which is another way to dampen inflammatory reactions [23]. The role of A20 in the negative inflammatory control is supported by multiple observations. In particular, A20-deficient mice are hyper-reactive to LPS and TNF-α, they develop spontaneous uncontrolled multi-organ inflammation and cachexia, and they die prematurely due to their incapacity to terminate the NF-κB response [24,25]. Mice bearing myeloid cell- specific *A20* deletion develop rheumatoid arthritis-like pathologies and enthesitis, and they display disruptions in myeloid cell hematopoietic precursors [18,20,26,27]. *A20*-deficient mouse macrophages demonstrate increased caspase-1 activation, and they over-produce TNF-α, IL-6, and IL-1β [18,26]. In humans, the haplo-insufficiency of *A20* leads to generalized inflammation [28,29,30,31]. *A20* single nucleotide polymorphisms have been associated with increased susceptibility to type 1 diabetes, Crohn’s disease, systemic lupus erythematosus (SLE), psoriasis, and systemic and multiple sclerosis [11,32,33,34,35]. Patients with these pathologies were reported to have a decreased level of the *A20* transcript in their blood [36,37,38]. The implication of A20 in inflammation control makes the protein a promising candidate for targeting exacerbated inflammatory reactions, particularly in macrophages [39]. However, most A20 studies were conducted in conditions wherein there was a deficit of A20. Whether the overexpression of the protein can efficiently restrict inflammatory reactions has been examined in a limited number of studies which did not involve macrophages [19,40,41,42,43,44,45,46] (this is considered in more detail in the Discussion section).

Macrophages derived from peripheral blood monocytes (MDMs) represent the most widely used model for studying human macrophage biology. However, due to their low proliferative activity and natural resistance to genetic modification, MDMs are difficult to genetically modify [47,48]. The generation of macrophages from induced pluripotent stem cells (iPSCs) allows to overcome the aforementioned limitation by introducing genetic modifications at the level of iPSCs and differentiating the latter into macrophages (iMacs). iMacs with stable genetic modifications of various genes have been successfully generated (reviewed in [49]). Whether it is possible to generate genetically modified iMacs with a controllable expression of a gene of interest has not been evaluated.

In this study, we used the CRISPR/Cas technology to generate iPSCs with tetracycline-inducible *A20* overexpression, and we elaborated a modified protocol of iPSC differentiation allowing the preservation of the tetracycline-inducible *A20* overexpression in differentiated iMacs. We presented new data on the dynamics of *A20* expression during iMac differentiation; for the first time, we documented a specific trait of macrophages consisting in the expression of a short (~70 kDa) form of the A20 protein, and we also showed the capacity of A20 overexpression to diminish the TNF-α-induced expression of *IL1B* and *IL6* proinflammatory cytokines at the mRNA level. The model elaborated in the study may be used to generate iMacs with a controllable expression of other genes of interest.

## 2. Results

### 2.1. A20-Transfected iPSCs Retain Pluripotent Properties

**Figure 1:**
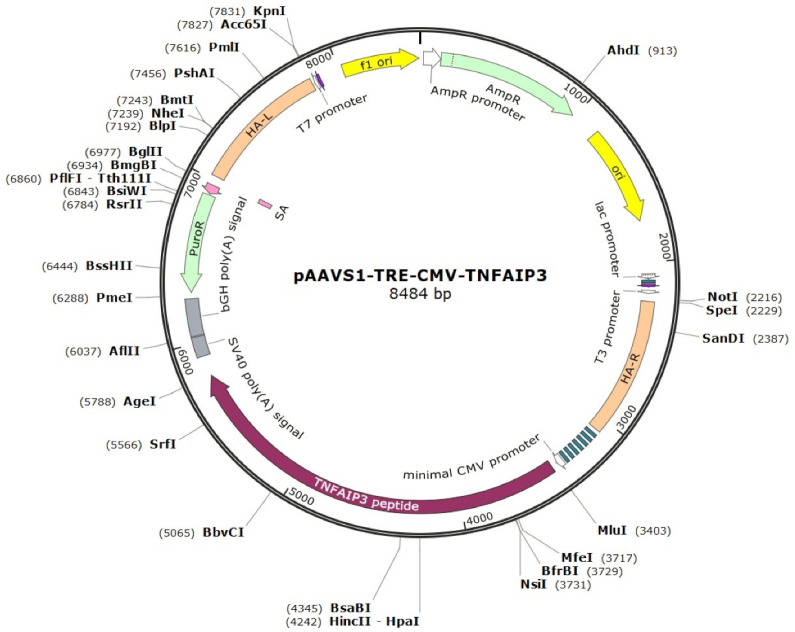
The scheme of the AAVS1-CMV-TRE-hTNFAIP3 donor plasmid containing the *A20* gene under a tetracycline-inducible promoter.

To generate an iPSC line with a tetracycline (doxycycline)-inducible expression of *A20* (A20-iPSCs)*,* we used the previously generated and characterized iPSC line K7-4Lf (hereafter referred to as K7-iPSCs) derived from human peripheral blood mononuclear cells (PBMCs) [50]. The cells were transfected with plasmid vectors encoding (i) gRNA/SpCas9 (pX458-AAVS1); (ii) reverse tetracycline transactivator (*M2rtTA*) and neomycin resistance gene (AAVS1-Neo-M2rtTA); and (iii) *A20* and puromycin resistance gene (Figure 1; see Section 4 for more details).

**Figure 2:**
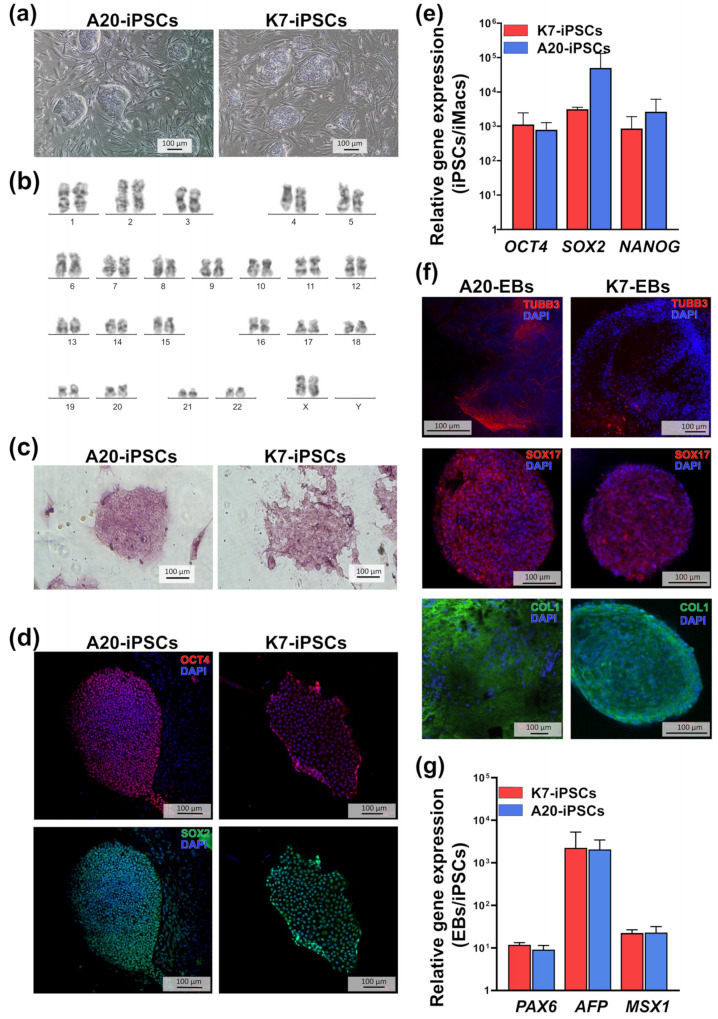
A20-iPSCs display the typical characteristics of pluripotent stem cells. (**a**) The morphology of A20-iPSC and K7-iPSC colonies. Light microscopy, phase contrast. (**b**) Cytogenetic analysis shows the normal karyotype of A20-iPSCs (46 XX). Numbers indicate chromosomes. (**c**) A20- and K7-iPSC colonies are positively stained for alkaline phosphatase. (**d**) A20- and K7-iPSCs express pluripotency markers OCT4 (red) and SOX2 (green). Confocal microscopy; nuclei are stained with 4′-6-diamidino-2-phenylindole (DAPI, blue). (**e**) A20- and K7-iPSCs express pluripotency-associated genes *OCT4*, *SOX2* and *NANOG* (RT-qPCR, gene expression in iPSCs relative to iMacs). (**f**,**g**) EBs spontaneously generated from A20- and K7-iPSCs contain cells expressing ectoderm, endoderm and mesoderm markers. (**f**) The expression of TUBB1 (ectoderm), SOX17 (endoderm) and COLI (mesoderm) visualized by immunostaining and confocal microscopy. (**g**) The expression of *PAX6* (ectoderm), *AFP* (endoderm) and *MSX1* (mesoderm) detected in RT-qPCR (the expression in EBs relative to iPSCs). All results were obtained in at least two independent experiments. For A20-iPSCs, the data obtained using the line A20-13 are shown. Similar results were obtained using the A20-24 line. Confocal microscopy: Zeiss LSM 880 (Carl Zeiss, Jena, Germany).

After neomycin/puromycin selection, four A20-iPSC clones with a confirmed target and the absence of off-target inserts of the donor plasmids were obtained. All of them displayed iPSC-like morphology, actively proliferated and “pushed away” the underlying feeder cells when growing (Figure 2a). Three of the four clones (i.e., A20.13, A20.9, and A20.24) had a normal 46 XX karyotype (Figure 2b). During the maintenance, the A20.13 iPSC line showed the highest growing capacity; therefore, it was used in most experiments (where indicated, the data were reproduced using the A20.24 line).

To assess the pluripotent potential of A20-iPSCs, we first stained them for alkaline phosphatase, a traditional marker of pluripotent stem cells [51,52]. We registered a homogeneously high-level expression of the enzyme that was comparable with that seen in the parental K7 line (Figure 2c).

Transcriptional factors OCT4 and SOX2 support cell pluripotency by sustaining cell self-renewal and restraining cell differentiation [53,54]. The expression of both factors by A20-iPSCs was shown by immunostaining (Figure 2d) and confirmed in RT-qPCR. RT-qPCR also demonstrated a high-level expression of another stem cell marker, *NANOG* [54,55] (Figure 2e). The levels of *OCT4, SOX2* and *NANOG* expression in A20-iPSCs were significantly (more than 10^3^-fold) higher compared with the expression levels of these genes in differentiated cells (i.e., in mature iMacs), which was reminiscent of the results obtained in the parental K7 line (Figure 2e).

To evaluate the tri-lineage differentiation potential of A20-iPSCs, we cultured them in low-adhesion conditions and analyzed spontaneously generated EBs for the expression of ectoderm (Tubulin β3, TUBB3), endoderm (SOX17) and mesoderm (Collagen I, COLI) markers. The expression of all three markers was confirmed by immunostaining (Figure 2f). Furthermore, RT-qPCR revealed a 9- to more than 1000-mean-fold increase in the expression of other ectoderm (Paired Box 6, *PAX6*), endoderm (Alpha Fetoprotein, *AFP*) and mesoderm (Msh Homeobox 1, *MSX1*) genes [56,57,58,59] in EBs compared with undifferentiated A20-iPSCs (Figure 2g).

Thus, *A20*-transfected iPSCs retained pluripotent cell properties.

### 2.2. A20-Transfected iPSCs Respond to Doxycycline by a Significant Increase in A20 Expression

**Figure 3:**
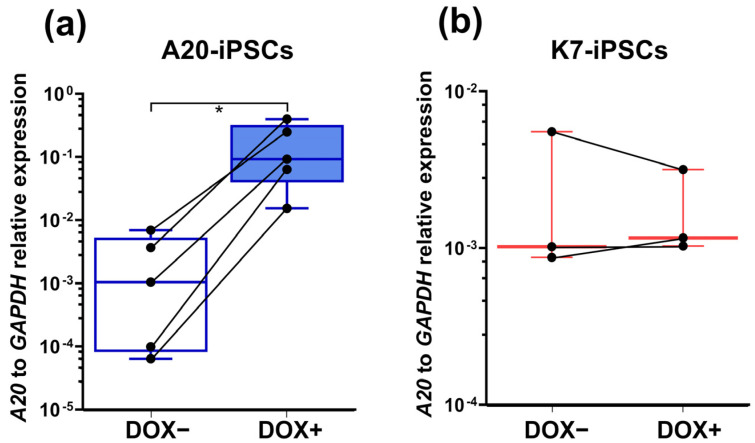
A20-iPSCs display DOX-inducible expression of *A20*. A20- and K7-iPSCs were cultured in the presence or absence of DOX for 24 h followed by the isolation of RNA and RT-qPCR. (**a**) A20-iPSCs stimulated with DOX overexpress *A20* compared with DOX-unstimulated cells (5 independent experiments, Mann–Whitney test, * *p* < 0.05). (**b**) In K7-iPSCs, DOX stimulation does not affect *A20* expression (3 independent experiments). As housekeeping genes, *GAPDH* and *ACTB* were used and provided similar results; the results shown are those obtained using *GAPDH*.

To verify the tetracycline-inducible expression of *A20* in A20-transfected iPSCs, we cultured A20-iPSCs in the presence of doxycycline (DOX) for 24 h and analyzed *A20* expression in RT-qPCR. In five independent experiments, treatment with DOX induced a significant upregulation of *A20* expression (98.5 (75.4 to 341.3)-fold, *p* = 0.002; Figure 3a). Similar results were obtained using the *A20*-iPSCs line A20.24. In parental K7-iPSCs, DOX stimulation did not affect *A20* expression (Figure 3b).

### 2.3. A20-Transfected iPSCs Can Be Efficiently Differentiated into iMacs

**Figure 4:**
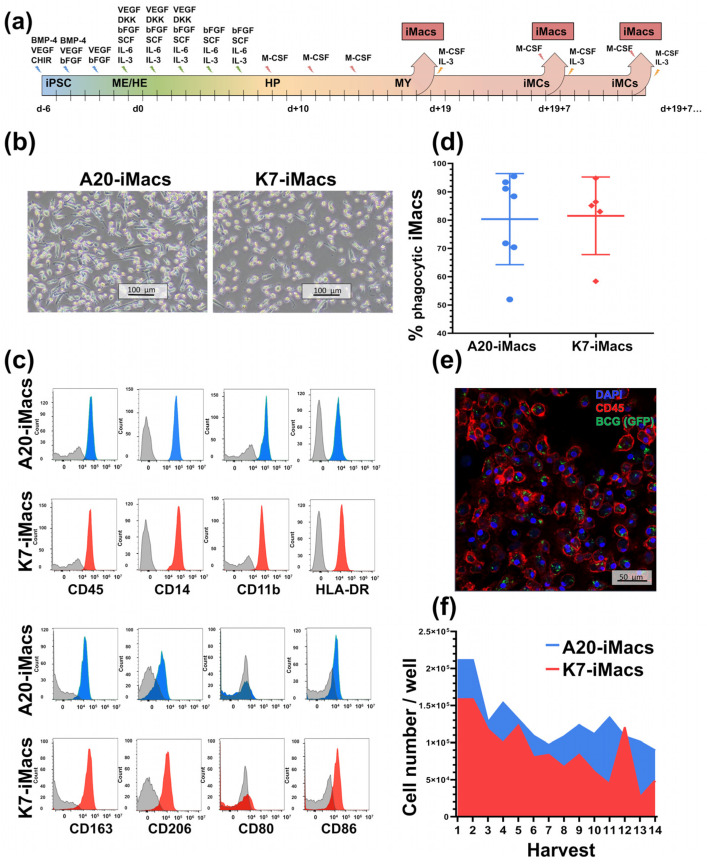
A20-iMacs differentiated from A20-iPSCs display cell morphology, phenotype and phagocytic activity typical for macrophages. A20-iPSCs and K7-iPSCs were differentiated into A20-iMacs and K7-iMacs in parallel using the protocol suggested by Takata and co-authors [60] (with our modifications [61]). (**a**) Schematic representation of the differentiation protocol. (**b**) Terminally differentiated A20- and K7-iMacs display cell morphology typical for macrophages. (**c**) A20- and K7-iMacs express CD11b, CD14 and CD45 and co-express markers of M1 and M2 macrophages (representative results of 5 independent experiments). (**d**) A20- and K7-iMacs display high phagocytic activity as determined in the Phagotest. Summarized results of 7 (A20-iMacs) and 5 (K7-iMacs) independent experiments. (**e**) A20- and K7-iMacs display high phagocytic activity as determined by cultivating the cells with GFP-expressing BCG. Representative data of two independent experiments (red, CD45; blue, DAPI; green, BCG; Zeiss LSM 880 confocal microscope). (**f**) Weekly yields of A20- and K7-iMacs. Blue, A20-iMacs; red, K7-iMacs. ME/HE, mesoderm/hemogenic endothelium formation stage; HP, hematopoietic specification; MY, myeloid specification; iMCs, monocyte-like cells.

After having confirmed the pluripotency state and the DOX-inducible expression of *A20* in A20-iPSCs, we differentiated them into iMacs (A20-iMacs) using the protocol published by Takata and co-authors [60] with our modifications [61] (Figure 4a). In parallel, K7-iPSCs were also differentiated into iMacs (K7-iMacs).

A20-iMacs had typical iMac morphology, i.e., they were large (~20 µm), round-shaped, equipped with pseudopodia and capable of plastic adherence (Figure 4b). The cells expressed CD45, CD11b and CD14 myeloid cell/macrophage markers and co-expressed the markers of M1 (CD86, HLA-DR) and M2 (CD163, CD206) macrophages, which corresponds to the previously reported characteristics of iMacs [62] (Figure 4c). In Phagotest, A20-iMacs displayed a high-level phagocytic activity (>80% phagocytic cells; Figure 4d). The latter was confirmed by the capacity of A20-iMacs to phagocyte GFP-expressing BCG (Figure 4e).

The morphology, phenotype and phagocytic activity of A20-iMacs were similar to those seen in K7-iMacs (Figure 4b–d). The productivity of iMac generation was also similar for A20- and K7-iMacs (Figure 4f). The results obtained with the A20-iPSC line A20.13 were confirmed using the A20.24 line.

Altogether, A20-transfected iPSCs could be successfully differentiated into iMacs that displayed typical macrophage characteristics.

### 2.4. Macrophages Differentiated from *A20*-Transfected iPSCs Lose DOX-Inducible A20 Expression, Which Can Be Fixed by Modifying the iMac Differentiation Protocol

**Figure 5:**
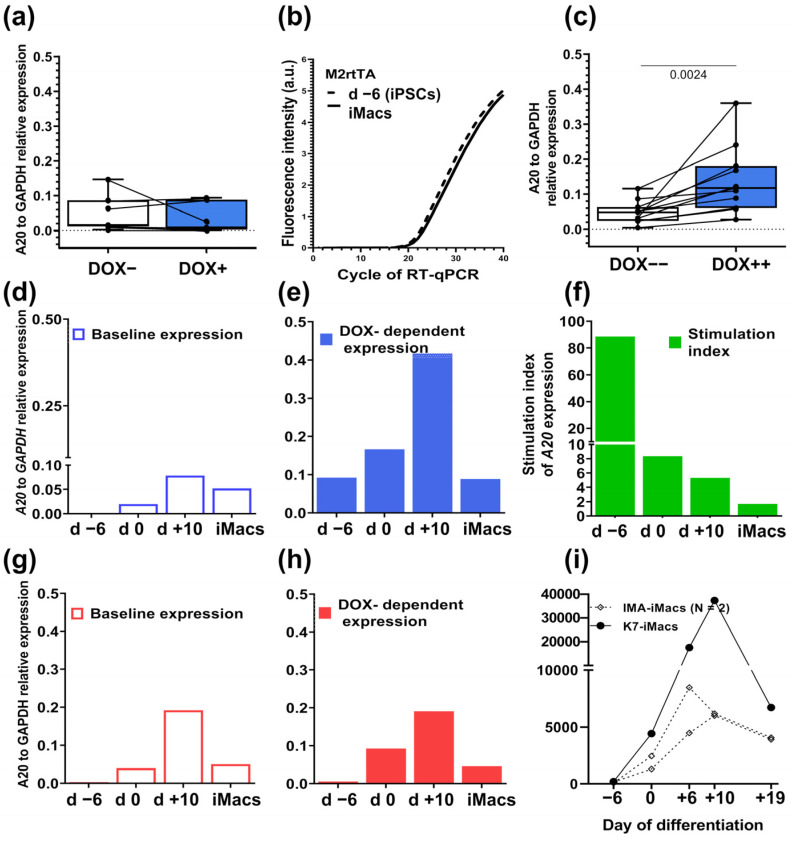
The levels of *A20* expression change following iPSC to iMac differentiation. A20- and K7-iPSCs were differentiated into A20- and K7-iMacs in parallel in the absence (**a**,**b**,**d**,**g**) or presence (**c**,**e**,**h**) of DOX. RNA was isolated from all differentiating cells (days −6, 0, +10) and from the resulting iMacs (day +19). *A20* expression was analyzed in RT-qPCR using *GAPDH* and *ACTB* as house-keeping genes. Shown is expression relative to *GAPDH*. (**a**), A20-iMacs differentiated in the absence of DOX and stimulated with DOX 24 h prior to RNA isolation lack DOX-inducible *A20* expression (summarized results of 7 independent experiments performed using the iPSC lines A20-13 and A20-24). (**b**) A20-iPSCs and A20-iMacs display similar levels of the expression of *M2rtTA* (RT-qPCR, the results of one representative experiment). (**c**) A20-iMacs differentiated in the presence of DOX reproducibly upregulate *A20* expression in response to DOX stimulation. DOX++, DOX was added to the cultures at each medium change during the differentiation and 24 h prior to RNA isolation from A20-iMacs; DOX- - cells were differentiated in the absence of DOX and were not stimulated with DOX 24 h prior to RNA isolation (summarized results of 11 independent experiments; Mann–Whitney test). (**d**,**e**) Baseline (**d**) and DOX-dependent (**e**) expression of *A20* at different stages of the differentiation of A20-iPSCs into A20-iMacs. (**d**) The cells were differentiated in the absence of DOX and were not stimulated with DOX prior to RNA isolation. (**e**) The cells were differentiated in the presence of DOX, and A20-iMacs were additionally stimulated with DOX 24 h prior to RNA isolation. (**f**) The stimulation index of *A20* expression calculated as the level of DOX-dependent expression divided by the baseline expression on the corresponding differentiation day. Representative results of one out of three independent differentiation experiments. (**g**,**h**) Baseline (**g**) and DOX-dependent (**h**) expression of *A20* at different stages of the differentiation of K7-iPSCs into K7-iMacs. (**i**) Normalized expression of *A20* gene as determined in RNA-seq performed at different stages following the differentiation of K7-iPSCs (one experiment) and iMA-iPSCs (two independent experiments) into iMacs (raw read counts available at Gene Expression Omnibus (GEO, NCBI) repository under accession GSE220450 [63].

After showing that *A20* transfection did not alter the iPSC capacity to differentiate into iMacs, we next checked whether A20-iMacs preserved the DOX-inducible expression of A20 seen in A20-iPSCs. Terminally differentiated A20-iMacs were stimulated with DOX for 24 h or left unstimulated; *A20* expression was assessed in RT-qPCR. Unexpectedly, we found no increase in *A20* expression in DOX-stimulated A20-iMacs compared with DOX-unstimulated ones, which indicated that the inducibility of *A20* expression was lost during the process of iMac differentiation (Figure 5a).

The Tet-On system used in our study to create *A20*-transfected iPSCs relies on the expression of M2rtTA transactivator and its DOX-dependent binding to tetO tetracycline operator positioned upstream of the minimal CMV promoter and the *A20* gene (Figure 1). RT-qPCR showed that *M2rtTA* was equally well transcribed in both A20-iPSCs and A20-iMacs (Figure 5b). Therefore, we supposed that the loss of the doxycycline-inducible *A20* expression in A20-iMacs was due to a loss of the tetO operator accessibility to M2rtTA, which could develop as a result of a permanent absence of DOX and a lack of promoter activity during the iMac differentiation process. We reasoned that the supplementation of differentiation media with DOX could help overcome the problem.

To test the hypothesis, we set up new experiments in which we differentiated A20-iPSCs into A20-iMacs in the presence of DOX and evaluated *A20* expression in (i) the resulting A20-iMacs (day +19, floating iMac population) and (ii) the differentiating cells (i.e., plastic-adherent population) at different stages of their differentiation (days −6, 0, +10, +19). Cells differentiated from A20-iPSCs and K7-iPSCs in the absence of DOX were used as controls (“baseline A20 expression”).

We found that culture supplementation with DOX during the differentiation process restored the capacity of A20-iMacs to upregulate *A20* in response to DOX, albeit at a lower level compared with A20-iPSCs (Figure 5c). Dynamic analysis revealed significant changes in both baseline and DOX-dependent A20 expression over the differentiation of A20-iPSCs.

The baseline *A20* expression was undetectable in iPSCs (day −6), gradually increased by differentiation day +10 (in three independent differentiation experiments, 92-, 76- and 56-fold) and declined in iMacs (7-, 1.5- and 4-fold compared with day +10), still remaining significantly higher compared with source iPSCs (13-, 50- and 14-fold; Figure 5d). The level of DOX-dependent *A20* expression was relatively high in iPSCs, gradually increased until day +10 (completion of hematopoietic and initiation of myeloid specification stage) and declined in iMacs (Figure 5e). The DOX stimulation index (SI) calculated as the level of DOX-dependent expression divided by the baseline expression on the corresponding differentiation day was at its maximum in iPSCs and declined following the differentiation, reaching its minimum in iMacs (Figure 5f). Apparently, the high SI seen in iPSCs was due to the extremely low level of the baseline *A20* expression in these cells.

During the differentiation of K7-iPSCs, a similar baseline dynamic expression of *A20* was registered, with its peak seen on day +10 (Figure 5g). Differentiation of K7-iPSCs in the presence of DOX did not significantly affect the levels of *A20* expression, indicating that DOX itself did not influence the expression of *A20* (Figure 5h).

Our results indicated that during iMac differentiation, A20 expression gradually increased following cell hematopoietic specification and declined in differentiated iMacs and that this pattern did not depend on *A20* transfection. To verify whether the pattern of *A20* expression was specific only to K7-iPSC line and its A20-iPSC derivatives or whether it is general in nature, we reanalyzed the results of our previous dynamic RNA sequencing performed during the differentiation of K7 and iMA iPSC lines into iMacs [61,63]. We found that similarly to the current results, during the differentiation of iMA-iPSCs, *A20* expression was extremely low in iPSCs and peaked at the stage of cell hematopoietic specification (days +6/+10; Figure 5i).

Overall, our analysis revealed a specific pattern of *A20* expression during iMac differentiation and demonstrated that the supplementation of the differentiation medium with DOX is a necessary and sufficient condition for preserving DOX-inducible *A20* expression in terminally differentiated A20-iMacs.

### 2.5. Macrophages Are Characterized by the Expression of a Low-Weight form of the *A20* Protein

**Figure 6:**
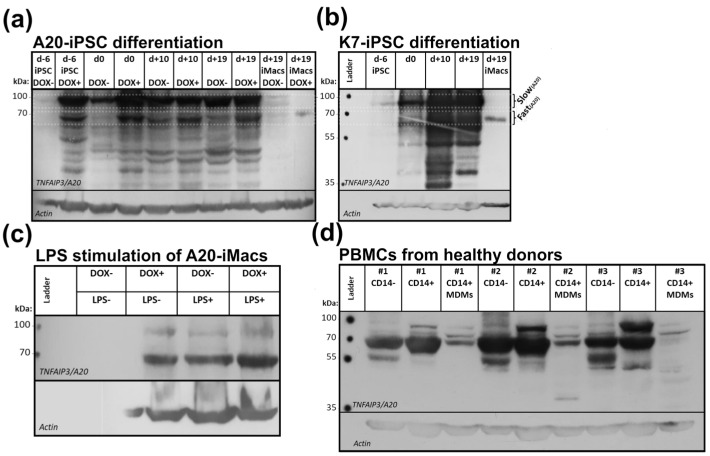
Macrophages express the A20 protein at a lower level and of lower molecular weight compared with less differentiated cells. (**a**,**b**) Following the differentiation of A20-iPSCs and K7-iPSCs, A20 expression peaks at the stage of hematopoietic specification and declines in iMacs. (**a**) A20-iPSCs were differentiated into A20-iMacs in the absence (DOX^−^) or presence (DOX^+^) of DOX. On differentiation days −6 (iPSCs), 0, +10 and +19, plastic-adherent differentiating cells were collected, pelleted and frozen for subsequent western blotting. For iMac analysis, on day +19, floating cells were collected and pelleted. To visualize A20 expression in A20-iMacs, the film was overdeveloped. (**b**), K7-iPSCs were differentiated in the absence of DOX and tested for A20 expression on the indicated days. Cells were collected as described in (**a**). (**c**) A20-iMacs stimulated with LPS express a 70 kDa form of the A20 protein. A20-iMacs were generated in the presence of DOX and stimulated with DOX, LPS or DOX+LPS or left unstimulated. The results of one out of two representative experiments are presented. (**d**) Similar to iMacs, MDMs are characterized by a low-level expression of A20 and a predominance of a 70 kDa form. PBMCs were isolated from three healthy donors, CD14^+^ and CD14^−^ populations were magnetically sorted, CD14^+^ cells were differentiated into MDMs, and all three populations were analyzed in Western blot.

To examine A20 expression following iMac differentiation at a protein level, we next performed Western blotting (Figure 6a,b). In differentiating (i.e., plastic-adherent) cells, the baseline A20 expression was undetectable on day −6 (iPSCs), became explicit on day 0 (mesoderm/hemogenic endothelium) and increased on days +10 and +19 (hematopoietic and myeloid specifications, respectively [64]). In iMacs (that were collected on day +19 as floating cells), A20 expression dropped and the bands were scarcely detectable (Figure 6a). Similar results were obtained during the differentiation of K7-iPSCs (Figure 6b).

In contrast to the baseline A20 expression, DOX-dependent expression was high in A20-iPSCs and in differentiating cells collected on days 0, +10 and +19; in iMacs it was barely visible and required a prolonged time of film development to be identified (Figure 6a).

It is worth noting that on SDS-electrophoresis, the A20 protein migrated as a stack of bands ranging from approximately 35 kDa to approximately 100 kDa. The most intense bands were those with a molecular weight of ~90 and ~70 kDa on which we then focused our attention. For ease of description, hereafter these bands are referred to as “slow-migrating” and “fast-migrating”, respectively. According to the Human Protein Atlas, the slow-migrating ~90 kDa form corresponds to the theoretically predicted full-size human A20 protein [65,66].

Unexpectedly, we noticed that while in iPSCs, day 0, day +10 and day +19 cells in a slow-migrating A20 form predominated, in DOX-stimulated A20-iMacs and in K7-iMacs, a fast-migrating A20 was more abundant (Figure 6a,b). We further verified these data by analyzing A20 expression in A20-iMacs stimulated with LPS, which is known to stimulate A20 expression [14,67]. A20-iMacs differentiated in the presence of DOX were left unstimulated or were stimulated with DOX, LPS or both stimuli. In DOX-unstimulated A20-iMacs, the A20 protein was barely detectable. However, the protein was identifiable in A20-iMacs stimulated with DOX, LPS or DOX plus LPS, and in all of these cells, a predominance of a fast-migrating form was detected (Figure 6c).

To check whether the predominant expression of a fast-migrating form of the 20 protein is a specific trait of iMacs or a general feature of macrophages, we then analyzed the A20 protein in MDMs. PBMCs were obtained from three healthy donors and sorted into CD14^+^ (monocytes) and CD14^−^ (predominantly, lymphocytes) populations. The former were differentiated into MDMs, and all three populations were subjected to Western blotting (Figure 6d). In all cells, a fast-migrating A20 form predominated. In the CD14^−^ population, besides a fast-migrating ~70 kDa band, a lower molecular weight band of the A20 protein was also identified. There were some inter-donor differences in the intensities and the ratio of the expression of ~70 kDa and ~90 kDa forms of the A20 protein. Given that all three donors were of the same sex (all women) and of similar age (22–36 years old), it is unlikely that the differences were sex- or age-related. However, additional studies are needed to explore whether and how age, gender and host inflammatory state affect the expression of different A20 protein forms. Of note, in all analyzed samples, MDMs displayed a significantly lower level of A20 expression compared with the monocyte population, which was reminiscent of iMacs.

Altogether, the experiments demonstrated, for the first time, that macrophages in general express the A20 protein at a lower level compared with less differentiated cells and that iMacs express an A20 protein of a lower molecular weight compared with their in vitro progenitors.

### 2.6. Overexpression of A20 Exhibits Different Effects on iMac Response to LPS and TNF-α

A20 deficiency exacerbates inflammatory reactions at cellular and organism levels [12,13,26,29]. We went on to examine whether our model of *A20* overexpression induced a degree of protection against macrophage inflammatory reactions. For that purpose, we analyzed baseline inflammatory state and the reactivity to LPS and TNF-α of DOX-treated and DOX-untreated A20-iMacs.

A20-iMacs were differentiated in the presence or absence of DOX, collected and cultured in a DOX-supplemented or DOX-free medium, respectively. Twenty-four hours later, LPS or TNF-α were added to the cultures or the cells were left unstimulated; the expressions of *IL1B*, *IL6* and *IL10* were analyzed 6 (LPS) or 18 (TNF-α) hours later.

**Figure 7:**
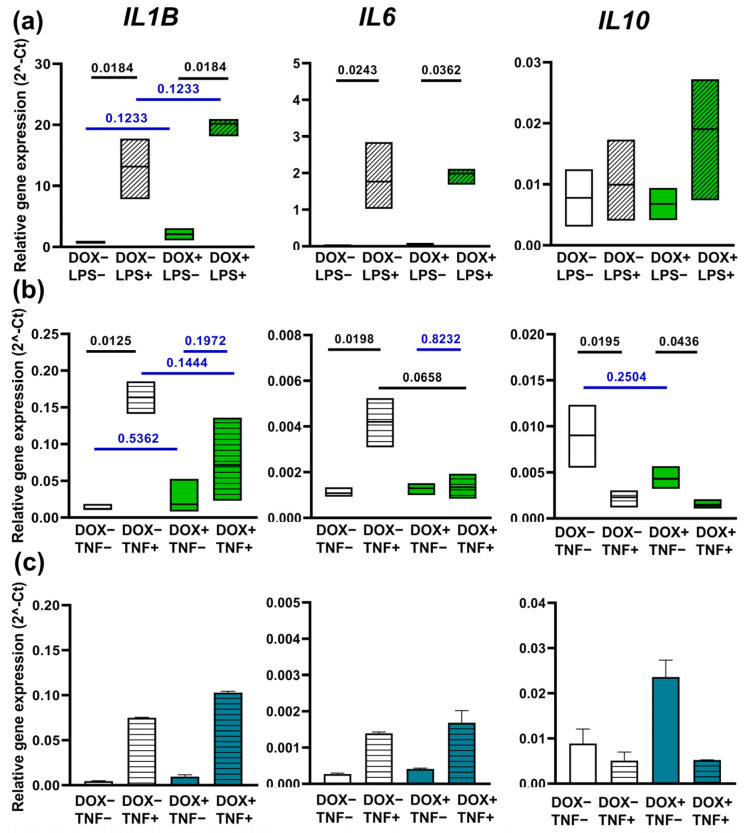
*A20* overexpression dampens TNF-α-elicited inflammatory response in A20-iMacs**.** A20-iMacs and K7-iMacs were differentiated in the presence or absence of DOX, treated (or not treated) with DOX 24 h before the stimulation and stimulated with LPS or TNF-α. The expression levels of *IL1B*, *IL6* and *IL10* were analyzed by RT-qPCR. (**a**) A20-iMacs respond to LPS by upregulation of *IL1B* and *IL6*. Note that treatment with DOX did not affect the level of cytokine expression in LPS-unstimulated and LPS-stimulated A20-iMacs. Summarized data of 2 independent experiments. (**b**) A20-iMacs respond to TNF-α by upregulation of *IL1B* and *IL6* and downregulation of *IL10*. Note that treatment with DOX abrogated TNF-α-induced upregulation of *IL1B* and *IL6.* Summarized data of 2 independent experiments. (**c**) K7-iMac response to TNF-α. Note that treatment with DOX did not influence the level of cytokine expression. The results of one representative experiment out of two are presented (the two experiments provided similar results in terms of the effects induced by DOX and TNF-α, but differed by the baseline levels of cytokine expression, which hindered their summarized analysis). (**a**,**b**) Median and interquartile intervals, Kruskal–Wallis test with Benjamini, Krieger and Yekutieli post-test for multiple comparisons. Shown are q-values; q > 0.1 are highlighted in blue (indicated where it is important to emphasize a lack of significant differences). (**c**), Median ± SD of technical replicates obtained in one out of two similar experiments.

The baseline expression of the cytokines was similar in DOX-treated and DOX-untreated A20-iMacs (Figure 7a, compare groups DOX^−^LPS^−^ and DOX^+^LPS^−^). In response to LPS, the expression of *IL1B* and *IL6* was significantly upregulated (q ≤ 0.0010). No significant differences between DOX-treated and DOX-untreated A20-iMacs in the magnitude of their reactivity to LPS were detected, indicating that *A20* overexpression did not mitigate A20-iMac response to LPS.

In contrast to LPS, A20-iMac response to TNF-α was affected by DOX. Whereas DOX-untreated A20-iMacs responded to TNF-α by a significant upregulation of *IL1B* and *IL6* (q < 0.02), pretreatment with DOX made the response insignificant (q = 0.1972 and q = 0.8232 for *IL1B* and *IL6*, respectively; Figure 7b). Of note, in K7-iMacs, the treatment with DOX did not impair TNF-α-induced response, evidencing that the effect seen in A20-iMacs was not due to a direct influence of DOX on inflammatory pathways, but was rather A20-mediated.

## 3. Discussion

We developed a model for generating human macrophages with tetracycline (DOX)-inducible expression of a gene of interest and to study the effects of *A20* overexpression in human macrophages. The model is based on the generation of *A20* overexpressing iPSC lines and their subsequent differentiation into iMacs using a protocol adjusted to sustain DOX-inducible *A20* overexpression during the cell differentiation process. To date, the feasibility of generating genetically modified macrophages by differentiating them from genetically modified iPSCs has been well documented. The scope of the studies includes model experiments [68], an introduction or a correction of disease-associated mutations [69,70,71,72,73,74], the analysis of the function of individual genes (e.g., *USP18* and *LRRK2* [75,76] and the generation of transgenic macrophages and myeloid cell lines for cancer therapy [77,78]. In all of the studies, a constitutive expression (or a loss of expression) of a target gene was introduced. However, having in mind the prospects of macrophage-based cell therapy [3], the development of approaches allowing the control (i.e., a chance to temporarily induce or silence) of transgene expression in macrophages is of great interest.

The A20-iPSC lines generated in this study harbor the *A20* transgene under the control of Tet-On (DOX-inducible) promoter in the “safe harbor” AAVS1 locus. The lines display all the major characteristics of pluripotent stem cells and respond to DOX by a significant increase in A20 expression at both mRNA and protein levels. However, A20-iMacs differentiated from A20-iPSCs lose the capacity to upregulate *A20* in response to DOX unless their differentiation is carried out in the presence of DOX. Our data are in line with findings made earlier by other groups that examined DOX-inducible expression of target genes in neurons. Specifically, Zhu and co-authors discovered that during the development of transgenic mice with rtTA expression, a stably integrated Tet-On promoter was silenced in mouse neurons, probably due to a decrease in chromatin accessibility for a tetracycline transactivator [79]. Ustyantseva and co-authors [80] showed that human motor neurons differentiated in vitro from iPSCs with a DOX-inducible expression of H_2_O_2_ biosensors lost biosensor expression, but a regular supplementation of the differentiation medium with DOX helped to overcome the problem. Our data extend these observations to iPSC-derived macrophages. Whether the silencing of a Tet-On promoter also occurs during the differentiation of other types of cells, besides macrophages and neurons, and whether it takes place only following cell differentiation from pluripotent stem cells (either induced or embryonic) or constitutes a more general trait of long-term culture procedures remains to be explored.

Examining the stage at which iMac differentiation decline in DOX-inducible A20 expression occurred in our model, we performed a dynamic analysis of *A20* expression during iPSC to iMac differentiation. We found a stage-specific pattern of a baseline A20 expression during iMac differentiation, which consists of a lack of A20 expression in iPSCs, a gradual increase in A20 expression from differentiation day 0 (mesoderm/hemogenic endothelium stage [64]) to day +10 (the end of hematopoietic specification) and a decline in iMacs. RT-qPCR results were confirmed by Western blotting. They were also supported by the results of our recent dynamic transcriptomic analysis of cells undergoing differentiation from two different iPSC lines to iMacs. Finally, a decline in the A20 protein expression was also observed in MDMs (compared with blood monocytes). We suggest that the biological meaning of the discovered pattern consists in the following. iPSCs display low-level expression of receptors implicated in the induction of inflammatory signaling (i.e., TLR2, TLR4, IL-1BR, IL6R etc. [61,63]); this makes negative inflammation control in iPSCs unnecessary and may explain under-expression of A20 in these cells. Following hematopoietic specification, the expression of inflammation-related receptors increases; accordingly, an elevated expression of A20 serves to prevent inflammatory reactions in immature cells that are not designated for inflammatory activity. Finally, macrophages are intended to mount inflammatory responses; in these cells, A20 expression declines, but it remains inducible and tangible enough to ensure the required level of inflammation control.

The model of macrophage differentiation used in our study recapitulates embryonic hematopoiesis [60,81]. Whether a similar dynamic of A20 expression takes place during adult hematopoiesis has not been directly explored. However, it has been demonstrated that in mice, full depletion of *A20* in hematopoietic stem cells (HSCs) and multipotent progenitors hampers the hematopoietic process and leads to the development of anemia and lymphopenia, a loss of HSC quiescence and an impairment of HSC reconstitution capacity [82,83]. Heterozygous *A20* deletion led to an inflammatory-like (aging-like) phenotype of hematopoietic stem and progenitor cells which manifested as their expansion, reduced fitness and myeloid-biased differentiation [84]. A20 deficiency in myeloid cells decreased the number of monocytes, monocyte-derived cells and common monocyte and granulocyte precursors and increased the density of microglia [27]. These results strongly indicate the importance of A20 for the maintenance of hematopoiesis and are in a good line with our data showing a peak of A20 expression at the stage of cell hematopoietic specification. Further direct dynamic analysis of A20 expression during adult hematopoiesis is ongoing and should shed more light on the role of the A20 protein in hematopoietic processes.

Another finding of this study is the identification of the two forms of the A20 protein, slow- and fast-migrating in immunoblot, in cells undergoing differentiation into iMacs. While in iPSCs and their hematopoietic progeny a slow-migrating ~90 kDa form predominated, in mature iMacs a fast-migrating ~70 kDa band prevailed (DOX^+^ A20-iMacs and K7-iMacs). The prevalence of a fast-migrating ~70 kDa form was also characteristic for blood cells, including CD14^+^ monocytes and MDMs. Thus, in our study, the predominance of a fast-migrating ~70 kDa form of the A20 protein appeared as a specific trait of immune cells, particularly mature macrophages. In the research literature, a direct comparison between the mobility of the A20 protein isolated from macrophages and that isolated from other hematopoietic or non-hematopoietic cells is missing. In those studies, where the expression in macrophages and blood cells was examined and where molecular-weight markers were indicated, different molecular weights of the A20 protein were reported, i.e., 70 kDa (B-cells from healthy donors and whole blood cells from patients with chronic lymphocytic leukemia [85], 82 kDa (mouse RAW264.7 cell line [86], and around 90 kDa (monocytes from healthy donors and patients with poly-autoimmunity [31], activated T-cells [28]).

The molecular basis for the differences between the A20 forms with different immunoblot mobility is not yet clear, and may include gene polymorphism, posttranslational modifications and peptide degradation. In their detailed study, Zammit and co-authors [87] described the presence of two forms of the A20 protein, slow- and fast-migrating (~90 and 75–80 kDa, respectively) in blood cells obtained from healthy donors. The predominant expression of one or another form depended on A20 polymorphism: the fast-migrating form predominated in healthy donors carrying heterozygous or homozygous T108A; I207L A20 allele; the slow-migrating form was predominant in noncarriers, it was S-381 phosphorylated and associated with a better inflammation control. Our results on the expression of slow- and fast-migrating A20 forms in human blood cells and iMac progenitors are in line with the data by Zammit and co-authors. However, in our study, the A20 transgene (Supplementary Table S3) did not carry mutations. Similarly, the analysis of the clinical exome of a patient who was a donor of the PBMCs that were used to obtain K7-iPSCs did not reveal a single nucleotide polymorphism in the coding region of the *A20* gene (only the intron polymorphism NM_001270508.2(TNFAIP3):c.805 + 28A > C with a benign status was found [88]). Thus, our data extend previous observations by demonstrating that the expression of the two forms of the A20 protein is a characteristic feature of human hematopoietic and blood cells and that one or another form may predominate independently of *A20* polymorphism but depending on the cell differentiation stage.

Besides mutations, posttranslational modifications including glycosylation, phosphorylation and peptide cleavage represent other possible explanations for the heterogeneity of the A20 protein forms seen in hematopoietic cells [39]. The cleavage of the A20 protein is well-documented for T-lymphocytes. In contrast to macrophages, T-lymphocytes constitutively express A20. Following TCR stimulation, the protein is cleaved by MALT1-Bcl-10 complex into N-terminal 50 kDa and C-terminal 37 kDa species; the latter retains E3 ligase activity but is unstable. This ultimately results in a loss of the A20 capacity to inhibit NF-κB and allows T-cell activation to occur [89,90]. Interestingly, Malinverni and co-authors [91] previously supposed that besides cleaving A20 into 50 kDa and 37 kDa species, MALT1 can also cleave the protein between ZF6 and ZNF7, leading to the formation of a ~75 kDa fragment. So far, the formation of a 75 kDa fragment has not been experimentally proven. However, the anti-TNFAIP3 antibody clone 59A426 used in our study was predicted to be able to recognize such a fragment. This suggests that the fast-migrating ~70 kDa A20 form identified in our study in macrophages can represent the A20 protein truncated by ZNF7.

In our study, A20-iMacs treated with DOX and overexpressing A20 limited *IL6* and *IL1B* mRNA expression triggered by TNF-α, but did not influence the LPS-induced response. The latter seems to contradict the existing data. Indeed, A20 is known as an effective negative regulator of intracellular signaling cascades initiated by various stimuli including LPS. However, most of our knowledge on A20 activity comes from the analysis of inflammatory responses and pathologies in conditions where there is a deficit of A20 [12,13,14]. The effects of A20 overexpression were explored in fewer studies and largely in cells other than macrophages. The reported effects include: an inhibition of the phosphorylation of p38 and c-JUN and an augmentation of the levels of IkB in intestinal epithelioid cells IEC-6 bearing an ectopic *A20* expression and stimulated with LPS or CpG [43]; a repression of retinoic acid-inducible gene I (RIG-1) and RIG-1-dependent antiviral activity and an inhibition of TIR-Domain-Containing-Adaptor-Molecule 1 (TRIF)-mediated activation of interferon-stimulated response element (ISRE), NF-κB and IFN-β promoter in HEK293 cells co-transfected with *A20* and its molecular targets [19,41]; an inhibition of IL-8 secretion by intestinal epithelial CaCo cells transfected with an A20-expressing plasmid and stimulated with Pam3CSK4 [42]; a downregulation of the expression of proinflammatory chemokines Ccl2, Cxcl1 and Cxcl10 in *A20*-transfected *Stat3*-knockout mouse embryo fibroblasts triggered with TNF-α [45]; a decrease in the expression of IL-6, IL-18 and TNF-α in primary neonatal rat cardiomyocytes transduced with an *A20*-expressing lentivirus [46]; a decreased production of IFN-γ and IL-17 by CD4^+^ T cells derived from patients with SLE and transfected with an *A20*-expressing plasmid [44]; and a decreased TNF-α-induced apoptosis in *A20*-overexpressing Jurkat cells [40]. Although the studies are consistent in terms of anti-inflammatory A20 effects, they do not concern macrophages, and it cannot be excluded that in different cells the nuances of A20 inhibitory activity may be different.

Another explanation for the insignificant influence of *A20* overexpression on the LPS-triggered response of A20-iMacs is that the level of *A20* overexpression reached in our study (~2–3-fold) was insufficient to block cell reactivity to such a strong stimulus as LPS. However, a similar level of A20 overexpression was achieved at least in some other studies, e.g., in CD4^+^ T cells transfected with an *A20*-expressing pReceiver-M12 plasmid [44]. Next, LPS itself is a strong inducer of *A20*. In our study, A20-iMacs triggered with LPS upregulated *A20* expression by more than 10-fold, which could mask the effects of DOX-inducible *A20* expression. Of note, in the aforementioned study by Zhao and co-authors, A20-transfected CD4^+^ T cells were derived from SLE patients and initially had significantly downregulated levels of *A20* expression [44]. Furthermore, A20 may differentially affect signaling pathways triggered by LPS and TNF-α. For example, in hepatocytes, A20 inhibited the activation of c-JUN but did not affect the activation of NF-κB [92]. Finally, as suggested above, the fast-migrating ~70 kDa form of A20 identified in our study in macrophages may represent ZNF7-truncated A20. As shown previously by Lin and co-authors, the truncation of the ZNF7 domain ablates the A20 inhibitory effect on IRF3- and NF-κB-mediated gene expression [19]. Thus, it is tempting to suppose that in macrophages, A20 is cleaved between ZNF6 and ZNF7, which limits A20 inhibitory effects and ensures macrophage preparedness for a quick response to danger signals.

To summarize, the current study has several outcomes. First, it develops a model and demonstrates the feasibility of generating human macrophages with tetracycline-inducible *A20* overexpression. Second, it identifies, for the first time, the peculiarities of A20 expression in human macrophages that may serve to ensure the baseline preparedness of macrophages for a quick response to danger signals. In particular, it demonstrates a low-level baseline expression of A20 and the predominance of a low-molecular-weight form of the A20 protein in human macrophages. Finally, the study suggests that A20 overexpression can mitigate the response of human macrophages to TNF-α, at least at an mRNA level. Examination of A20 effects on a wide range of inflammatory cytokines and chemokines at a protein level, identification of molecular pathways involved in the A20 anti-inflammatory effect, and a more detailed analysis of the peculiarities of A20 expression in macrophages will be a subject of further studies. Overall, the results contribute to a better understanding of how macrophage inflammatory reactivity is regulated and suggest a novel model that can be used to study the effects of A20 and other target genes on macrophage functionality.

## 4. Materials and Methods

### 4.1. Reagents and Media

The suppliers and catalogue numbers of all materials are presented in Supplementary Table S1.

iPSCs were maintained in an iPSC medium consisting of a KnockOut™ DMEM medium supplemented with a 15% KnockOut™ Serum Replacement, 0.1 mM of non-essential amino acids, 1% penicillin/streptomycin, 1 mM GlutaMAX-I, 0.055 mM β-mercaptoethanol, and 10 ng/mL bFGF. For iMac differentiation, the following media were used: mTeSR™1 supplemented with 1% penicillin/streptomycin; StemPro-34™ supplemented with 5 mM of ascorbic acid, 200 ug/mL of human transferrin, 1% penicillin/streptomycin, 2 mM of GlutaMAX-I, 0.45 mM of monothioglycerol; RPMI-1640 supplemented with 2 µM of GlutaMAX-I, 1% non-essential amino acids, 1% penicillin/streptomycin, 0.055 mM of β-mercaptoethanol, 10% fetal calf serum and X-VIVO™15 supplemented with 2 mM of GlutaMAX-I and 1% penicillin/streptomycin.

### 4.2. iPSC Line Generation and Maintenance

The iPSC line K7-4Lf was previously generated from human PBMCs [93] and maintained as described earlier [50]. Briefly, the cells were cultured on mouse embryo fibroblast feeder cells (MEFs) in an iPSC medium until they reached a 70–80% confluence. For passages, cell colonies were disrupted using TrypLE and transferred to new MEF-coated wells at a 1:10–1:20 ratio. The medium was replaced daily; for the first 24 h of culture, 10 µM of Roc-kinase inhibitor (Y-27632) or 2 µM of Thiazovivin was added to the cultures. Cell viability was monitored periodically using Trypan blue staining.

### 4.3. Generation of iPSC Lines with Tetracycline-Controllable A20 Expression

The A20 encoding nucleotide sequence was obtained by PCR from pEGFP-C1-A20 (Addgene #22141) using a Q5^®^ High-Fidelity DNA Polymerase and primers (Supplementary Table S2). Plasmid assembly was performed using standard molecular cloning methods by replacing the MluI-AsiGI fragment in the pCyto-roGFP2-Orp1-donor plasmid [80] with the *A20* PCR product. The pAAVS1-TRE-CMV-TNFAIP3 construct assembly was confirmed by restriction analysis and Sanger sequencing (SB RAS Genomics Core Facility, Novosibirsk, Russia). Plasmid DNA was isolated using a PureLink™ HiPure Plasmid Midiprep Kit (Supplementary Table S1).

K7-iPSCs were passaged on MEF-coated Petri dishes one day before the electroporation. On the next day, the cells were dissociated using TrypLE, counted using a Countess™ automated cell counter (Thermo Fisher Scientific, CA, USA), and 10^6^ cells were transfected with an equimolar mix of the three donor plasmids (5 µg total) using the Neon™ Transfection System 100 μL Kit (Thermo Fisher Scientific) (one pulse, 30 ms, 1100 V). The following plasmids were used for K7-iPSC electroporation: (i) pX458-AAVS1 encoding guide RNA for human AAVS1 locus and SpCas9 protein [80]; (ii) AAVS1-Neo-M2rtTA (Addgene #60843) containing a constitutive M2rtTA expression cassette and neomycin resistance gene; and (iii) the generated pAAVS1-CMV-TRE-hTNFAIP3 plasmid containing *A20* gene cDNA under a tetracycline-inducible promoter (Supplementary Table S1; Figure 1).

The cells were then transferred into MEF-coated Petri dishes in penicillin/streptomycin-free iPSC medium supplemented with 10 mM of Y-27632. After small iPSC colonies were formed, puromycin and neomycin resistant clones were selected by culturing the cells sequentially in the presence of puromycin dihydrochloride (300 ug/mL, 3 days) and geneticin (50 µg/mL, 4–5 days). The grown clones were expanded. Genomic DNA was extracted from all clones using an QuickExtract™DNA Extraction Solution and analyzed for the presence of the target and off-target inserts of the donor plasmids (Supplementary Table S2).

### 4.4. Histochemical and Immunofluorescent Characterization and Karyotyping of iPSC Lines with Tetracycline-Controllable A20 Expression

The pluripotency of A20-iPSCs was tested by detecting alkaline phosphatase, analyzing the expression of pluripotency markers and evaluating the cell capacity to differentiate into the 3 germ layers.

For alkaline phosphatase staining, the cells were treated with the Alkaline Phosphatase kit according to the manufacturer protocol. Briefly, the cells were washed with 10 mM PBS, fixed in citrate–acetone–formaldehyde fixative, washed, stained with the Alkaline solution (15 min in the dark), washed with deionized water, counterstained with the Hematoxylin solution (2 min) and analyzed using an Olympus IX 51 microscope (Olympus, Shinjuku, Tokyo, Japan).

To assess the expression of pluripotency markers, A20-iPSCs were fixed (4% paraformaldehyde, 15 min), washed, permeabilized (1% Triton-X100, 60 min), washed, incubated in the Blocking Buffer (2.5% bovine serum albumin in phosphate-buffered saline (10 mM PBS); 30 min), treated with primary mouse-anti-OCT4 (1:200) and rabbit-anti-SOX2 (1:100) antibodies, washed and incubated with anti- mouse Alexa Fluor 568 (1:500) and anti-rabbit Alexa Fluor 488 (1:1000) secondary antibodies (room temperature, 90–120 min). Cell nuclei were stained with 4’-6-diamidino-2-phenylindole (DAPI).

To test the A20-iPSC differentiation potential, cell colonies were treated with the collagenase IV solution, removed with a scraper and cultured in low adhesion conditions for 21 days to allow spontaneous generation of embryoid bodies (EBs). The formed EBs were fixed (4% paraformaldehyde, 15 min), washed (3 times, 20 min each), permeabilized (1% Triton-X100, 60 min), blocked (1% bovine serum albumin in 10 mM PBS, 60 min) and incubated with mouse-anti-TUBB3 (ectoderm, 1:100), rabbit-anti-SOX17 (endoderm, 1:100) and/or rabbit-anti-COL1 (mesoderm, 1:50; 4 C, overnight). The EBs were then washed and incubated with Alexa 568 anti-mouse (1:500) or Alexa 488 anti-rabbit antibodies (1:200, room temperature, 90–120 min). Cell nuclei were stained with DAPI. The signals were visualized using the Zeiss LSM 880 confocal microscope (Carl Zeiss, Jena, Germany).

G-banding was performed as previously described [94]; karyotyping was performed at the Tomsk National Research Medical Center of the Russian Academy of Sciences using the International System for Human Cytogenetic Nomenclature. A total of 50 metaphase plates were analyzed using a Carl Zeiss Axioplan 2 imaging microscope and Ikaros karyotyping software V5.5 (Metasystems GmbH, Altlussheim, Germany).

### 4.5. iPSC Differentiation into iMacs

The differentiation of iPSCs into iMacs was performed using the previously described method [60] with our modifications [61]. Briefly, iPSCs were depleted of MEFs, disaggregated with collagenase IV, collected and cultured in Matrigel-coated 6-well plates in a supplemented StemPro-34 medium (differentiation day −6). Until culture day +10, the medium was changed every other day; besides standard supplements, it was supplemented with the following factors: day −6: CHIR99021 (an inhibitor of glycogen synthase kinase 3; 2 mM), bone morphogenetic protein 4 (BMP4; 5 ng/mL), vascular endothelial growth factor A (VEGFA; 50 ng/mL); day −4: BMP4 (5 ng/mL), VEGFA (50 ng/mL), basic fibroblast growth factor (bFGF; 20 ng/mL); day −2:, VEGFA (15 ng/mL), bFGF (5 ng/mL); days 0, +2, +4: VEGFA (10 ng/mL), bFGF (10 ng/mL), Dickkopf-related protein 1 (DKK1; 30 ng/mL), stem cell factor (SCF; 50 ng/mL), IL-6 (10ng/mL), IL-3 (20 ng/mL); days +6 and +8: bFGF (10 ng/mL), SCF (50 ng/mL), IL-6 (10 ng/mL), IL-3 (20 ng/mL) (Figure 2). Starting on day +10, the StemPro-34 medium was substituted for the supplemented RPMI-1640, which contained a macrophage colony-stimulating factor (M-CSF; 50 ng/mL). The medium was changed every 3 days until day +19. On day +19, floating cells were collected (first iMac harvest). The remaining adherent cells were cultured in X-VIVO™15 medium supplemented with IL-3 (25 ng/mL) and M-CSF (50 ng/mL). Seven days later, floating monocyte-like precursors of iMacs appeared in the cultures; they were collected, counted and terminally differentiated into a new portion of iMacs (12- or 6-well plates; 1.5–2.5 10^5^ cells/mL density; supplemented RPMI-1640 medium containing 100 ng/mL M-CSF; 7-day culture). The original wells were re-stimulated with supplemented X-VIVO™15 or RPMI-1640 media containing IL-3/M-CSF to continue new rounds of the generation of monocyte-like precursors and mature iMacs.

### 4.6. Cell Treatment with Doxycycline

DOX was dissolved in water at a 2 mg/mL concentration, sterilized by filtration through a 0.22 µm filter and stored at –20 °C until the day of use but no longer than for 1 month. If not indicated otherwise, DOX was added to iPSCs or iMacs 24 h prior to the experiments. In iMac, differentiation experiments performed in the presence of DOX, DOX was added to the culture medium each time the medium was changed.

### 4.7. Real-Time qPCR

RNA was isolated using the RNeasy Micro (for cell numbers < 10^5^) or RNeasy Mini (cell numbers > 10^5^) kits and QIAshredder columns according to the manufacturer’s instructions. RNA concentrations were determined using a NanoDrop One spectrophotometer (Thermo Fischer Scientific, Waltham, MA, USA). Reverse transcription was performed using the Maxima First Strand cDNA Synthesis Kit for RT-qPCR. Within each experiment, cDNAs were prepared using the same amount of RNA. Real-time qPCR was performed using the HS TaqDNA Polymerase dNTP mix kit, primers and probes listed in Supplementary Table S2 and LightCycler 480 Real-Time PCR System (Roche, Basel, Switzerland). The results were analyzed using the LightCycler^®^ 96 program. Ct values were normalized to *ACTB* and *GAPDH*.

### 4.8. Western Blotting

For Western blotting, 2–3 10^6^ cells were washed in 10 mM of PBS, pelleted and stored at –80 °C. To obtain whole-cell extracts, the pellets were treated with an RIPA Lysis and an Extraction Buffer in the presence of a cOmplete™ protease inhibitor cocktail, and centrifuged (12,000× *g*, 20 min, 4 °C). Protein concentration was measured using a Pierce BCA Protein Assay Kit according to the manufacturer’s instructions. A total of 30 µg of total proteins was electrophoresed on a 12% SDS/polyacrylamide gel (90 V followed by 180–190 V [95], transferred onto a 0.45 µm nitrocellulose NitroPure™ membrane, blocked with a 5% non-fat dry milk in a TNT buffer (10 mM of Tris-HCl, pH 7.5, 150 mM of NaCl, 0.1% Tween-20) and incubated with primary mouse anti-human-A20 (1:500) or mouse anti-human actin-β (1:10,000) monoclonal antibodies dissolved in a TNT buffer with a 1% milk (4 °C, overnight). The membranes were then incubated with horseradish peroxidase-conjugated polyclonal goat anti-mouse IgG (2 h, room temperature). Chemiluminescence was detected using an ECL Western Blotting Detection Kit. X-ray films were scanned and the resulting images were processed with the standard ImageJ 1.53a software for evaluating the integral absorption of protein bands. Normalization was performed using β-actin content per lane.

### 4.9. Flow Cytometry

Cells (1–3 10^5^) were stained with the following antibodies: FITC-anti-CD80, PE-anti-CD206, PerCP-Cy5.5-anti-CD14, APC-anti-HLA-DR and BV711-anti-CD163 or FITC-anti-CD45, PerCP-Cy5.5-anti-CD14 and Alexa700-anti-CD11b or Alexa700-anti-CD86. The cells were washed and analyzed on a CytoFLEX-S cytometer (Beckman Coulter, Brea, CA, USA) using the CytEXPERT software version 2.4.0.28 (Beckman Coulter). Unstained and single-stained controls were used for instrument settings. For gating, isotype controls and a single-stained anti-CD14 control were used (preliminary experiments showed that single-stained anti-CD14 control provided a level of background staining similar to that of various fluorescence-minus-one FMO controls. The results were analyzed using the FlowJo^TM^ software v10.8.1 (TreeStar BD Bioscience, Franklin Lakes, NJ, USA).

### 4.10. Phagocytosis Assays

Phagocytosis was assessed using the commercial Phagotest™ kit according to the manufacturer’s instructions. Briefly, 3∙10^5^ cells were resuspended in 200 μL of 10 mM of PBS supplemented with a 50% fetal calf serum. All samples were placed on ice for 10 min. FITC-labeled *E. coli* bacteria were added, and the cells were incubated at 37 °C (test sample) or 0 °C (negative control) for 30 min. The assay was stopped by adding 100 μL of a quenching solution; the samples were fixed, washed, treated with PerCP-Cy5.5-anti-CD14 antibodies and analyzed by flow cytometry. As an additional test, the iMac capacity to phagocyte GFP-expressing mycobacteria *M. bovis* BCG was evaluated. iMacs (10^5^ cells/well) were co-cultured with GFP-BCG (2 × 10^5^ bacteria/well; a kind gift of Dr. Nelli F. Khabibullina) in 24-well Cell Imaging Plates for 3 days; the cells were stained with anti-CD45-Alexa700 antibodies and analyzed using an Axio Scope A1 microscope.

### 4.11. The Generation of Monocyte-Derived Macrophages

Venous blood was collected from three healthy donors (all women; median age 25 years; range 22–36 years) in EDTA tubes. PBMCs were isolated on a Ficoll gradient (density 1.077 g/mL; 600 g, 15 min, room temperature). After being washed twice (Dulbecco’s PBS without Ca and Mg, 600 and 300 g, 15 min, room temperature), PBMCs were magnetically sorted into CD14^−^ and CD14^+^ populations using human CD14 MicroBeads following the manufacturer’s instructions. The CD14^−^ population and an aliquot of the CD14^+^ population were washed twice, pelleted and frozen for subsequent Western blotting analysis. The remaining CD14^+^ monocytes were cultured in a supplemented RPMI-1640 medium containing 100 ng/mL of M-CSF for MDM generation. MDMs were prepared for Western blotting as described above. The studies were approved by the Institutional Review Board of the Koltzov Institute of Developmental Biology of RAS (protocol No. 63 of 20 October 2022).

### 4.12. Cell Stimulation with LPS and TNF-α

Terminally differentiated iMacs were collected, counted and cultured in 12-well plates at 2∙10^5^ cells/mL in the presence or absence of DOX. Twenty-four hours later, the cells were stimulated with *E. coli* LPS (a smooth-form LPS from the Gram-negative bacteria *E. coli* 055: B5; 100 ng/mL, 6 h) or TNF-α (10 ng/mL, 18 h) or left unstimulated. At the end of cell culture, RNA was isolated for RT-qPCR.

### 4.13. Statistical Analysis

Most data are shown as medians and interquartile [25%; 75%] ranges; normally distributed data (e.g., Phagotest results) are shown as mean ± SD. Differences between the groups were analyzed using the non-parametric Kruskal–Wallis test with the post hoc Mann–Whitney test; for multiple comparisons, the false discovery rate (q-value) was controlled using the two-stage step-up method of Benjamini, Krieger and Yekutieli (GraphPad Prizm 8.0; GraphPad Inc., San Diego, CA, USA). *p*-value and q-value < 0.05 were considered statistically significant.

## Data Availability

The datasets generated for this study are available on request to the corresponding author.

## Supplementary Materials

The following supporting information can be downloaded at: https://www.mdpi.com/article/10.3390/ijms241612868/s1.

## Abbreviations

bFGF: basic fibroblast growth factor; BMP4, bone morphogenetic protein 4; DAPI, 4’-6-diamidino-2-phenylindole; DKK1, Dickkopf-related protein 1; DOX, doxycycline; EBs, embryoid bodies; M-CSF, macrophage colony-stimulating factor; MEFs, mouse embryo fibroblast feeder cells; M2rtTA, reverse tetracycline transactivator; PBMCs, peripheral blood mononuclear cells; PBS, phosphate-buffered saline; PCR, polymerase chain reaction; RT-qPCR, quantitative reverse transcription polymerase chain reaction; SCF, stem cell factor; SI, stimulation index calculated as the level of DOX-dependent expression divided by the baseline expression on the corresponding differentiation day; SLE, systemic lupus erythematosus; VEGFA, vascular endothelial growth factor A.
